# Stress Causing Factors Among Teachers in Elementary Schools and Their Relationship with Demographic and Job Characteristics

**DOI:** 10.3889/oamjms.2015.077

**Published:** 2015-07-01

**Authors:** Teuta Agai–Demjaha, Jordan Minov, Sasho Stoleski, Beti Zafirova

**Affiliations:** 1 *Institute of Occupational Health of Republic of Macedonia, WHO Collaborating Center, Department of Occupational Medicine, Medical Faculty, Ss Cyril and Methodius University of Skopje, Skopje, Republic of Macedonia*; 2 *Institute of Epidemiology and Biostatistics with Medical Informatics, Medical Faculty, Ss Cyril and Methodius University of Skopje, Skopje, Republic of Macedonia*

**Keywords:** workplace, stress causing factors, teachers, elementary school, job characteristics

## Abstract

**BACKGROUND:**

Once high levels of work-related stress among teachers were confirmed many studies concentrated on identifying and investigating key stress factors among school teachers. Unfortunately there are very few researches made on stress causing factors among teachers in Republic of Macedonia.

**AIM:**

To determine the most frequent stress causing factors among teachers in elementary schools and to investigate their relationship with demographic and job characteristics.

**METHODOLOGY:**

We performed a descriptive-analytical model of a cross-sectional study which involved 300 teachers employed in nine elementary schools. Evaluation of examined subjects included completion of a specially designed questionnaire.

**RESULTS:**

Among six categories of factors that generate work related stress (job demands, control, relationships, role, changes and support) control and support had the highest mean scores. Within the control category the highest levels of perceived teacher’s work-related stress were caused by the following factors - changes in terms and conditions without consultation and given responsibility without the authority to take decisions. 141 out of the interviewed teachers (47%) have mentioned changes in terms and conditions without consultation as very stressful, while another 50 (16.67%) have reported it as stressful. 123 out of interviewed teachers (41%) have stated given responsibility without the authority to take decisions as very stressful, with another 105 (35%) have reported it as stressful. In the category support the highest levels of perceived teacher’s work-related stress were caused by stress factors - lack of funds/resources to do the job and limited or no access to training. Out of 300 interviewed teachers, 179 (59.67%) have reported lack of funds/resources to do the job as very stressful, while another 50 (16.67%) as stressful. There is no significant relationship between the stress factor limited or no access to training and demographic and job characteristics.

**CONCLUSION:**

Our findings confirm that within the control category, the highest levels of perceived teacher’s work-related stress were caused by changes in terms and conditions without consultation and given responsibility without the authority to take decisions, while in the category support, the same was true for stress factors lack of funds/resources to do the job and limited or no access to training. We have also concluded that the lower-grade school teachers, female teachers, teachers for whom this is the first job and teachers with university education perceive more often the lack of authority to take decisions as a very stressful factor than the upper-grade school teachers, male teachers, teachers previously employed in another workplace, and those with high education. The lower-grade school teachers, older teachers and teachers with university education perceive more often changes in education as a very stressful factor than the upper grade school teachers, younger teachers and those with high education.

## Introduction

Education undoubtedly represents one of the most critical sectors when speaking of work-related stress. Work related stress among teachers was firstly identified during 1930s in a study by Smith and Milstein [1]. Later on in 1955, Travers and Cooper have documented the history of changes in the educational process as well as the impact of these changes on teachers’ stress [2]. In their daily jobs teachers increasingly faced tasks and demands that generated work-related stress and reduced job productivity [3-6]. Moreover, a comparative study by Johnson S. et al. in 2005 concluded that out of 26 professions, teaching represents the second most stressful occupation after ambulance car drivers [7].

Once high levels of work-related stress among teachers were confirmed, many studies concentrated on identifying and investigating key stress factors among school teachers.

According to a study by Pettigrew and Wolf, conducted in 1982, there are two types of stress which might ultimately have an impact on teachers: stress based on work related tasks and stress based on the workplace role. Stress based on work related tasks, such as *dealing with misbehaving students*, addresses the problems associated with different specific tasks that teachers must perform as part of their occupation and job description. Stress based on the workplace role such as *lack of necessary resources for proper teaching*, refers to how the expectations of teachers about their role in the workplace fit with their real responsibilities that are necessary for teachers to fulfil their roles [8].

Often, stress within teaching is connected with organizational factors related to the way teachers are expected to work. Such organizational factors that contribute to stress among teachers can be: unreasonably set time frames, excessive bureaucracy, unrealistic deadlines and frightening inspection regimes [9]. The causes of stress among teachers can also be defined as stressors of living and working environment as well as individual stressors. Most stressors are associated with the working environment and include unfavourable working conditions, excessive workloads, organizational problems, and insufficient resources, lack of support and/or autonomy, and decision making. The working environment may also include physical stressors such as noise associated with teaching assignments, accrued classrooms, size of the classroom and/or school, security and violence among youth as well as administrative pressures such as lack of support from managers and ambiguity of the teaching role. Individual characteristics include the unique attributes of teachers such as personality, gender, age, demographics, ability to establish and maintain supportive networks, cognitive evaluation of stressors, coping ability, type of teaching position and work dissatisfaction [10, 11]. Individual stress, can also be linked to the compatibility between personal and educational values, ambition to succeed, the threshold of sensitivity, competitiveness, multiple roles for women teachers (parent, caretaker, housewife and teacher), and perfection [12]. One of the few analyses in the field of work-related stress in Macedonia is the comparative study of Eres and Atanasovska that explores the levels of stress among teachers in Turkey and their colleagues in Macedonia. Their study suggests that working conditions as well as personal and social characteristics may have an effect on teacher stress [13].

The aim of this paper was to determine the most frequent stress causing factors among teachers in elementary schools and to further investigate their relationship with demographic and job characteristics (gender, age, position in the workplace, the current job being the first job and the level of education).

## Methodology

### Study design and setting

The research was carried out in nine elementary schools in Skopje, Republic of Macedonia by using a descriptive-analytical model of an epidemiological cross-sectional study. Prior to the research, ethical approval was granted by the Ministry of Education and Science. Evaluation of examined subjects included completion of a specially designed questionnaire. Out of 358 teachers to whom the questionnaire was distributed, 300 of them responded. The interviewing process was voluntary and respected the teachers’ rights to anonymity. The research was conducted in close cooperation with experts from the Institute of Occupational Health of RM, WHO Collaborating Center and it took place between September 2013 and June 2014.

### Subjects

The research included 300 teachers from elementary schools, aged from 26 to 64 years. Out of the overall number of subjects, 195 (65%) were females and 105 (35%) were males. According to the research data, 45% of the subjects were lower-grade school teachers, while 55% worked as upper-grade school teachers. In terms of education, 159 (53%) of teachers had high education,^1^ while 141 (47%) were with university (superior) education. Out of overall number of subjects, 174 (58%) stated that the current job was their first job while 126 subjects (42%) declared to have been previously employed.

### Questionnaire

The questionnaire concerning the impact of stress on teachers’ health in elementary schools has been used as an instrument of the study. It was based on the UCU (University and College Union) Model Stress Questionnaire^2^ and it consists of five parts. For the purpose of this paper we have utilized part one of the questionnaire which includes: data regarding the demographic and job characteristics of the respondents (gender, age, position in the workplace, the current job being the first job and the level of education) and part four which includes six different categories of factors that generate work - related stress (job demands, control, relationships, role, changes and support). Our results have shown that out of six analyzed categories of stress factors, *control* and *support* have the highest mean scores. Accordingly, more detailed results for work - related stress factors among teachers have been presented only for these two categories.

It should be mentioned that the category *control* has been analyzed through six main stress factors: (1) not able to exert control over demands made; (2) dealing with competing demands – unable to plan working day; (3) work linked to deadlines and targets; (4) changes in terms and conditions without consultation; (5) job changes without consultation and (6) given responsibility without the authority to take decisions. On the other hand, the category *support* has been analyzed through seven main stress factors: (1) lack of information about what is going on; (2) insufficient admin support; (3) lack of management support; (4) limited or no access to training; (5) over competitive/confrontational institutional culture; (6) lack of funds/resources to do the job; and (7) lack of equipment (photocopiers, etc.)

### Statistical methods

Statistical Package for the Social Science (SPSS) version 17.0 for Windows was used for data description and analysis. Categorical variables were expressed as absolute and relative number. The Chi-square test was used to test differences in relation to the different demographic and job characteristics. P-value below 0.05 was considered statistically significant while p-value below 0.01 was considered highly significant.

## Results

The research results based on data collected are presented below. Results are presented in tables and figures and also include additional textual description. Demographic and job characteristics of the respondents such as gender, age, position in the working place, the current job being the first job and the level of education are presented in Table 1.

**Table 1:** Demographic/job characteristics of the study subjects

Variable	N=300
Gender	
Female	195 (65%)
Male	105 (35%)
Age	
Under 45	83 (27.66%)
45 +	217 (73.34%)
Position in the workplace	
Teacher-lower grade	135 (45%)
Teacher-upper grade	165 (55%)
The current job is the first job	
Teacher-first job	174(58%)
Teacher-not first job	126 (42%)
Level of education	
High	159 (53%)
University (superior degree)	141 (47%)

Note: numerical data are expressed as mean value with standard deviation; frequencies as number and percentage of study subjects with certain variable.

**Figure 1:**
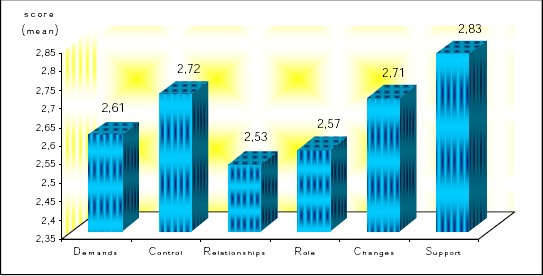
*Overall mean scores of six categories of stress factors*.

The different work related stress factors among teachers in our questionnaire have been divided in six main categories: (a) demands; (b) control; (c) relationships; (d) role; (e) changes; and (f) support. Overall mean scores for these different six categories of stress factors based on the questionnaire are shown in Figure 1.

According to our results, among the six analyzed categories, *control* and *support* have the highest mean scores. Therefore, more detailed results for work-related stress factors among teachers of these two categories and their relationship with demographic and job characteristics, are presented below:

### Control

Overall levels of teacher’s work-related stress for six stress factors within the category *control* are shown in Table 2.

**Table 2:** Levels of teacher’s work-related stress factors - category control

Which factors associated with your current post do you think create work related stress?	Not applicable	Occasionally stressful	Stressful	Very stressful
Not able to exert control over demands made	89 (29.67%)	57 (19%)	66 (22%)	88 (29.33%)
Dealing with competing demands – unable to plan working day	34 (11.33%)	97 (32.33%)	103 (34.33%)	66 (22%)
Work linked to deadlines and targets	84 (28%)	65 (21.67%)	76 (25.33%)	75 (25%)
Changes in terms and conditions without consultation	45 (15%)	64 (21.33%)	50 (16.67%)	141 (47%)
Job changes without consultations	79 (26.33%)	90 (30%)	45 (15%)	86 (28.67%)
Given responsibility without the authority to take decisions	52 (17.33%)	20 (6.67%)	105 (35%)	123 (41%)

According to the Table 2, among the six analyzed factors, the highest levels of perceived teacher’s work-related stress within the category ***control*** were caused by *changes in terms and conditions without consultation* and *given responsibility without the authority to take decisions*. Namely, out of 300 interviewed teachers, 141 (47%) have mentioned *changes in terms and conditions without consultation* as very stressful; while another 50 (16.67%) have reported it as stressful. On the other hand, 123 (41%) of interviewed teachers have stated *given responsibility without the authority to take decisions* as very stressful, with another 105 (35%) mentioning it as stressful.

The relationship of the gender, age, position in the working place, the current job being the first job and the level of education with the stress factor *changes in terms and conditions without consultation* is shown in Table 3.

**Table 3:** The relationship of demographic/job characteristics with the stress factor Changes in terms and conditions without consultation

Variable	Changes in terms and conditions without consultation			
	Not applicable	Occasionally stressful	Stressful	Very stressful
**Gender**				
Female	21 (10.77%)	47 (24.1%)	34 (17.44%)	93 (47.69%)
Male	24 (22.86%)	17 (16.19%)	16 (15.24%)	48 (45.71%)
Pearson chi-square: 1.14, df = 2, p = 0.57				
**Age**				
Under 45	18 (21.69%)	33 (39.76%)	19 (22.89%)	13 (15.66%)
45 +	27 (12.44%)	31 (14.29%)	31 (14.29%)	128 (58.99%)
Pearson chi-square: 46.68, df = 2, p = 0.000000**, p < 0.01				
**Position in the working place**				
Teacher Lower-grade	11 (8.15%)	24 (17.78%)	18 (13.33%)	82 (60.74%)
Teacher Upper-grade	34 (20.61%)	40 (24.24%)	32 (19.39%)	59 (35.76%)
Pearson chi-square: 11.49, df = 2, p = 0.003**, p < 0.01				
**The current job is the first job**				
Yes	40 (22.99 %)	16 (9.2%)	24 (13.79%)	94 (54.02%)
No	5 (3.97%)	48 (38.1%)	26 (20.63%)	47 (37.3%)
Pearson chi-square: 31.16, df = 2, p = 0.00000017**, p < 0.01				
**Level of education**				
High	39 (24.53%)	21 (13.21%)	23 (14.47%)	76 (47.8%)
University	6 (4.26%)	43 (30.5%)	27 (19.15%)	65 (46.1%)
Pearson chi-square: 7.89, df = 2, p = 0.019*, p < 0.05				

Note: frequencies are given as number and percentage of study subjects with certain variable.

* Tested by chi-square test.

According to our results, low-grade teachers consider the stress factor *changes in terms and conditions without consultation* more often as very stressful as compared to the upper-grade teachers (60.74% vs. 35.76%). The same finding was detected for teachers older than 45, as compared to those younger than 45 (58.99% vs. 15.66%), as well as for teachers for whom this is the first job compared to the teachers that have been previously employed (54.02% vs. 37.3%). On the other hand, teachers with university education perceive such stress factor more often as occasionally stressful and stressful as compared to teachers with high education - (30.5% vs. 13.21%) and (19.15% vs. 14.47%) respectively.

The relationship of the gender, age, position in the working place, the current job being the first job and the level of education with the stress factor *given responsibility without the authority to take decisions* is shown in Table 4.

**Table 4:** Relationship of demographic/job characteristics with the stress factor given responsibility without the authority to take decisions

Variable	Given responsibility without the authority to take decisions			
	Not applicable	Occasionally stressful	Stressful	Very stressful
Gender				
Female	24 (12.31%)	12 (6.15%)	64 (32.82%)	95 (48.72%)
Male	28 (26.67%)	8 (7.62%)	41 (39.05%)	28 (26.67%)
Pearson chi-square: 7.83, df = 2, p = 0.02*, p < 0.05				
Age				
Under 45	25 (30.12%)	5 (6.02%)	17 (20.48%)	36 (43.37%)
45 +	27 (12.44%)	15 (6.91%)	88 (40.55%)	87 (40.09%)
Pearson chi-square: 5.44 df = 2, p = 0.06				
Position in the working place				
Teacher Lower-grade	24 (17.78%)	11 (8.15%)	30 (22.22%)	70 (51.85%)
Teacher Upper-grade	28 (16.97%)	9 (5.45%)	75 (45.45%)	53 (32.12%)
Pearson chi-square: 19.32, df = 2, p = 0.00006**, p < 0.01				
The current job is the first job				
Yes	36 (20.69%)	13 (7.47%)	44 (25.29%)	81 (46.55%)
No	16 (12.7%)	7 (5.56%)	61 (48.41%)	42 (33.33%)
Pearson chi-square: 13.93, df = 2, p = 0.0009**, p < 0.01				
Level of education				
High	37 (23.27%)	1 (0.63%)	49 (30.82%)	72 (45.28%)
University	15 (10.64%)	19 (13.48%)	56 (39.72%)	51 (26.17%)
Pearson chi-square: 20.19, df = 2, p = 0.00004**, p < 0.01				

Note: frequencies are given as number and percentage of study subjects with certain variable.

* Tested by chi-square test.

The lower-grade teachers perceive the stress factor *given responsibility without the authority to take decisions* as very stressful more often than upper-grade school teachers (51.85% vs. 32.12%), and the same finding was detected for female teachers as compared to their male colleagues (48.72% vs. 26.67%). The results also show that teachers for whom this is the first job consider this stress factor as very stressful more often than teachers that have been previously employed (46.55% vs. 33.33%), as well as teachers with university education as compared to teachers with high education (45.28% vs. 26.17%).

### Support

Overall levels of teacher’s work-related stress for seven stress factors within the category *Support* are shown in Table 5.

**Table 5:** Levels of teacher’s work-related stress factors - category support

Which factors associated with your current post do you think create work-related stress?	Not applicable	Occasionally stressful	Stressful	Very stressful
Lack of information about what is going on	9 (3%)	108 (36%)	81 (27%)	102 (34%)
Insufficient admin support	41 (13.67%)	116 (38.67%)	60 (20%)	83 (27.67%)
Lack of management support	71 (23.67%)	61(20.33%)	70 (23.33%)	98 (32.67%)
Lack of funds/resources to do the job	42 (14%)	29 (9.67%)	50 (16.67%)	179 (59.67%)
Over competitive/confrontational institutional culture	75 (25%)	74 (24.67%)	57(19%)	94 (31.33%)
Limited or no access to training	68 (22.67%)	38 (12.67%)	39 (13%)	155 (51.67%)
Lack of equipment (photocopiers, etc.)	38 (12.67%)	47(15.67%)	50 (16.67%)	163 (54.33%)

Note: frequencies are given as number and percentage of study subjects with certain variable.

According to the Table 5, among the seven analyzed factors, the highest levels of perceived teacher’s work-related stress within the category ***support*** were caused by *lack of funds/resources to do the job* and *limited or no access to training*. Our data suggest that, out of 300 interviewed teachers, 179 (59.67%) have reported *lack of funds/resources to do the job* as very stressful, while another 50 (16.67%) as stressful. On the other hand, 155 (51.67%) of the interviewed teachers have reported *limited or no access to training* as very stressful, and another 39 (13%) consider this factor as stressful.

The relationship of the gender, age, position in the working place, the current job being the first job and the level of education with the stress factor *lack of funds/resources to do the job* is shown in Table 6.

**Table 6:** Relationship of demographic/job characteristics with the stress factor Lack of funds/resources to do the job

Variable	Lack of funds/resources to do the job			
	Not applicable	Occasionally stressful	Stressful	Very stressful
Gender				
Female	35 (42.17%)	19 (9.74%)	37 (18.97%)	104 (53.33%)
Male	7 (3.23%)	10 (9.52%)	13 (12.38%)	75 (71.43%)
Pearson chi-square: 4.36, df = 2, p = 0.1				
Age				
Under 45	35 (17.95%)	6 (7.23%)	13 (15.66%)	29 (34.94%)
45 +	7 (6.67%)	23 (10.6%)	37 (17.05%)	150 (69.12%)
Pearson chi-square: 2.57, df = 2, p = 0.3				
Position in the working place				
Teacher Lower-grade	27 (20%)	20 (14.81%)	17 (12.59%)	71 (52.59%)
Teacher Upper-grade	15 (9.09%)	9 (5.45%)	33 (20%)	108 (65.45%)
Pearson chi-square: 10.38, df = 2, p = 0.0055**, p < 0.01				
The current job is the first job				
Yes (%)	11 (6.32%)	11 (6.32%)	21 (12.07%)	131 (75.29%)
No (%)	31 (24.6%)	18 (14.29%)	29 (23.02%)	48 (38.1%)
Pearson chi-square: 25.29, df = 2, p = 0.000003**, p < 0.01				
Level of education				
High	15 (9.43%)	2 (1.26%)	25 (15.76%)	117 (73.58%)
University	27 (19.15%)	27 (19.15%)	25 (17.73%)	62 (43.97%)
Pearson chi-square: 35.44, df = 2, p = 0.00000002**, p < 0.01				

Note: frequencies are given as number and percentage of study subjects with certain variable.

* Tested by chi-square test.

In the case of the stress factor *limited or no access to training*, the results were somewhat different, with upper grades teachers considering it as a very stressful more often than the lower grades teachers (65.45% vs 52.59%). On the other hand, teachers for whom this is the first job also perceived this stress factor considerably more often as very stressful as compared to their colleagues who have been previously employed, (75.29% vs 38.1%), as well as teachers with high education as compared to teachers with university education (73.58% vs 43.97%).

The relationship of gender, age, position in the working place, the current job being the first job and the level of education with the stress factor *limited or no access to training* is shown in Table 7.

**Table 7:** Relationship of demographic/job characteristics with the stress factor Limited or no access to training

Variable	Limited or no access to training			
	Not applicable	Occasionally stressful	Stressful	Very stressful
Gender				
Female	37 (18.97%)	24 (12.31%)	30 (15.38%)	104 (53.33%)
Male	31 (29.52%)	14 (13.33%)	9 (8.57%)	51 (48.57%)
Pearson chi-square: 1.9, df = 2, p = 0.39				
Age				
Under 45	32 (38.55%)	18 (21.69%)	18 (21.69%)	15 (18.07%)
45 +	36 (16.59%)	20 (9.22%)	21 (9.68%)	140 (64.52%)
Pearson chi-square: 41.25, df = 2, p = 0.000000**, p < 0.01				
Position in the working place				
Teacher Lower-grade	29 (21.48%)	19 (14.07%)	18 (13.33%)	69 (51.11%)
Teacher Upper-grade	39 (23.64%)	19 (11.52%)	21 (12.73%)	86 (52.12%)
Pearson chi-square: 0.37, df = 2, p = 0.8				
The current job is the first job				
Yes	32 (18.39%)	26 (14.94%)	28 (16.09%)	88 (50.57%)
No	36 (28.57%)	12 (9.52%)	11 (8.73%)	67 (53.17%)
Pearson chi-square: 3.96, df = 2, p = 0.14				
Level of education				
High	50 (31.45%)	13 (8.18%)	18 (11.32%)	78 (49.06%)
University	18 (12.77%)	25 (17.73%)	21 (14.89%)	77 (54.61%)
Pearson chi-square:3.19, df = 2, p = 0.2				

Note: frequencies are given as number and percentage of study subjects with certain variable.

* Tested by chi-square test.

According to our results, the work related stress as a result of the stress factor *limited or no access to training*, did not significantly depend from different demographic/job characteristics, such as gender and age of the respondents, the position in the working place, the current job being the first job and the level of education.

## Discussion

Our research aimed to determine the most frequent stress causing factors among teachers in elementary schools and to investigate their relationship with demographic and job characteristics.

A study by Dlamini, Okeke and Mammen from 2014 has indicated that badly planned changes were a major source of work - related stress among teachers in Swaziland [14]. Changes in education as a major factor among sources of work - related stress among teachers were also reported by studies conducted in South Africa and Zimbabwe in 2002 [15, 16]. These studies indicate that teachers do not object to the changes themselves, but more to the manner in which they were implemented and the fact they were made without prior consultations. Our results are very much in line with these finding since 141 (47%) of the interviewed teachers have mentioned *changes in terms and conditions without consultation* as very stressful, while another 50 (16.7%) have reported it as stressful. However, contrary to our results, these studies did not find any important correlation between demographic/job characteristics and changes in education as a stress factor. Our study, on the other hand, has indicated that the lower- grade school teachers, older teachers and teachers with university education perceive more often changes in education (*changes in* *terms and conditions without consultation)* as a very stressful factor than the upper-grade school teachers, younger teachers and those with high education.

Neverthelss, a study by Nayak from 2008 has reported that a relatively high percentage of teachers (22%) always experienced stress due to lack of their involvement in decision making in their organization despite given responsibilities [17]. Our study supports these findings, though in our case, a much higher percentage of teachers have indicated stress factor *given responsibility without the authority to take decisions* as stressful. Namely, according to our results, 123 (41%) of interviewed teachers have stated *given responsibility without the authority to take decisions* as very stressful, with another 105 (35%) mentioning it as stressful. A study by Check & Okwob from 2012 has analyzed the correlation of demographic/job factors with different stress factors among teachers in Cameroon [18]. While the study indicated that non - involvement in decision making concerning teaching and learning is perceived by teachers as relatively stressful, it found no evidence of significant correlation between gender, level of education and other demographic/job factors, with this specific stress factors. Our study contradicts such findings since the lower-grade school teachers, female teachers, teachers for whom this is the first job and teachers with university education perceive more often the *lack of authority to take decisions (given responsibility without the authority to take decisions)* as a very stressful factor than the upper-grade school teachers, male teachers, teachers previously employed in another workplace, and those with high education.

Brown, Ralph and Brember on their study from 2002 have reported that teachers indicate lack of adequate funding for job implementation as source of their work - related stress [19]. Similarly, a study by Betrabet from 2012 reported that not having adequate resources for necessary and purposive action as a teacher represents a major stress factor [20]. In addition, a report of the European Trade Union Committee for Education (ETUCE) Survey on Teachers’ Work - related Stress in 2007 has indicated that *lack of funds/resources to do the job* represents a major stress factor among both lower grades and upper school teachers in all 27 countries under consideration [21]. Such findings are obviously supported by our study since out of 300 interviewed teachers, 59.7% have reported *lack of funds/resources to do the job* as very stressful, while another 16.7% as stressful. While our results showed that upper-grade school teachers, teachers for whom this is the first job and teachers with high education perceived more often *lack of funds/resources to do the job* as a very stressful factor than the lower-grade school teachers, teachers who have been previously employed and teachers with university education – there were no available studies that report such correlation between this stress factor and demographic/job characteristics.

A study by Öztürk from 2011 has reported different results regarding self - perceived work-related stress among Stockholm and Istanbul teachers in relation to the lack of opportunity for professional development/training [22]. Namely, while more than half 51% of Istanbul teachers consider this factor as stressful, none of their Swedish colleagues shared their opinion. Our results are almost identical with findings about Istanbul teachers, since 51.7% of the interviewed teachers have reported *limited or no access to training* as very stressful, and another 13% as stressful. Similarly to our study, other studies, including the one by Öztürk, have not found significant relationship between the stress factor *limited or no access to training* and different demographic/job characteristics such as the position in the workplace, gender and age of the respondents, the level of education and the current job as the first employment.

Relatively small number of the interviewed subjects represents a certain limitation of the study that could have certain implications on the obtained data and their interpretation. The same is also true for the fact that only teachers from nine schools in one urban municipality were interviewed. On the other hand, main strength of the study is that it represents one of few public health studies in the country that deal with the work-related stress factors in general, and the first one that analyses stress causing factors among teachers in particular.

In a cross-sectional study aimed to investigate main work - related stress factors among teachers in elementary schools and to determine the relationships between teachers’ stress and their demographic and job characteristics, we have concluded that among six analysed categories, *control* and *support* had the highest mean scores. Within the *control* category, we have concluded that the highest levels of perceived teacher’s work-related stress were caused by *changes in terms and conditions without consultation* and *given responsibility without the authority to take decisions*. In the category *support*, the same was true for stress factors *lack of funds/resources to do the job* and *limited or no access to training*. In terms of relationship between different stress factors and demographic/job characteristics, our conclusion was that the lower-grade school teachers, older teachers and teachers with university education perceive more often *changes in education* as a very stressful factor than the upper-grade school teachers, younger teachers and those with high education. Furthermore, it may be concluded that the lower-grade school teachers, female teachers, teachers for whom this is the first job and teachers with university education perceive more often *the lack of authority to take decisions* as a very stressful factor than the upper-grade school teachers, male teachers, teachers previously employed in another workplace, and those with high education. Our results also showed that upper-grade school teachers, teachers for whom this is the first job and teachers with high education perceive more often *lack of funds/resources to do the job* as a very stressful factor than the lower-grade school teachers, teachers who have been previously employed and teachers with university education. In addition, we have found no significant relationship between the stress factors *limited or no access to training* and different demographic/job characteristics.

On the other hand, it should be mentioned that the educational system in Republic of Macedonia has been undergoing serious and high paced reforms. The highest levels of perceived teacher’s work-related stress in Macedonia are caused by factors that are imposed by these changes. Coupled together, these two facts send a message to both teachers and policy makers that for successful and well received educational reforms, field of education has to be a stress-relieved environment for all actors involved in these changes. Finally, although a number of results from this study might be important for the potential they have in terms of public health and policy implications, it is clear that further research in this field remains an obvious necessity.

## Footnotes

1 Two year college education for receiving teacher’s degree after completing the high school. Within the undergoing educational reforms in R. Macedonia this degree has to be upgraded into a university degree.

2 For more details see http://www.ucu.org.uk/2562.
